# Wheat-*Thinopyrum* Substitution Lines Imprint Compensation Both From Recipients and Donors

**DOI:** 10.3389/fpls.2022.837410

**Published:** 2022-04-15

**Authors:** Zhongfan Lyu, Yongchao Hao, Liyang Chen, Shoushen Xu, Hongjin Wang, Mengyao Li, Wenyang Ge, Bingqian Hou, Xinxin Cheng, Xuefeng Li, Naixiu Che, Tianyue Zhen, Silong Sun, Yinguang Bao, Zujun Yang, Jizeng Jia, Lingrang Kong, Hongwei Wang

**Affiliations:** ^1^State Key Laboratory of Crop Biology, College of Agronomy, Shandong Agricultural University, Taian, China; ^2^Smartgenomics Technology Institute, Tianjin, China; ^3^Center for Informational Biology, School of Life Science and Technology, University of Electronic Science and Technology of China, Chengdu, China; ^4^Institute of Crop Sciences, Chinese Academy of Agricultural Sciences (CAAS), Beijing, China

**Keywords:** wheat, *Thinopyrum*, substitution line, compensation, distant hybridization

## Abstract

Even frequently used in wheat breeding, we still have an insufficient understanding of the biology of the products *via* distant hybridization. In this study, a transcriptomic analysis was performed for six *Triticum aestivum*-*Thinopyrum elongatum* substitution lines in comparison with the host plants. All the six disomic substitution lines showed much stronger “transcriptomic-shock” occurred on alien genomes with 57.43–69.22% genes changed expression level but less on the recipient genome (2.19–8.97%). Genome-wide suppression of alien genes along chromosomes was observed with a high proportion of downregulated genes (39.69–48.21%). Oppositely, the wheat recipient showed genome-wide compensation with more upregulated genes, occurring on all chromosomes but not limited to the homeologous groups. Moreover, strong co-upregulation of the orthologs between wheat and *Thinopyrum* sub-genomes was enriched in photosynthesis with predicted chloroplastic localization, which indicates that the compensation happened not only on wheat host genomes but also on alien genomes.

## Introduction

Broadening genetic diversity in wheat represents an indispensable step for any viable breeding strategy that aimed at tackling obstacles posed by environmental stress or plateaus in yield (Feuillet et al., 2008; Brozynska et al., 2016). In addition to intraspecific breeding using different wheat varieties, wild relative species of *Triticeae* are often deployed as alternative genetic resources for wheat improvement through distant hybridization. For example, translocation lines of the wheat-rye 4R and 6R show strong resistance to powdery mildew (Kubaláková et al., 2002; An et al., 2013, 2015). Wheat-*Thinopyrum* substitution lines harboring *Fhb7*, *Lr19*, and *Sr25* exhibited disease resistance to *Fusarium* head blight (FHB), leaf rust, and stem rust (Wang et al., 2020). Some superior genes were conferred by wheat-rye 1BL/1RS translocation lines, involved in high yield potential and biotic and abiotic stress tolerance, which promoted the production (Villareal et al., 1991; Mago et al., 2002; Lelley et al., 2004; Mohammed et al., 2013; Qi et al., 2016; Yang et al., 2016).

Currently, we still have insufficient understanding of the biology of the hybrid products by chromosome engineering. A long-standing stereotyped presumption is that large chromosomal segments/haplotypes from wheat relative species inherently contain deleterious alleles which often lead to poor performance of yield traits (Kerber and Dyck, 1973; Chen et al., 2005; Hurni et al., 2014; Stirnweis et al., 2014; Rey et al., 2018). However, it becomes increasingly clear that it is not the mere introgression of alien chromatin or genes in host; instead, it often transgresses the expected allele additivity in many aspects, i.e., genetic, epigenetic, and gene regulation, that may cause restructuring of transcriptome, proteome, metabolome, and phenome (Lynch and Conery, 2000; Prince and Pickett, 2002; Adams et al., 2003; Duarte et al., 2006; Buggs et al., 2011). With the deepening of research, studies also reported that the interaction between the donor and recipient genomes also affects plant phenotype (Chaudhary et al., 2009). For example, the interaction between pathogen-resistant gene and the recipient genome leads to inhibition of gene expression (Kerber and Dyck, 1973; Kerber and Aung, 1999; Chen et al., 2005). The formation of heteromeric complexes, which including donor and recipient gene products, may also cause harmful effects (Hurni et al., 2014). Therefore, further mechanistic understanding of distant hybridization-incurred “transcriptomic-shock” phenomenon will enhance our maneuvers (Buggs et al., 2011).

Wheatgrasses of *Thinopyrum* genus are well-known for employment in wheat distant hybridization (Ceoloni et al., 2017; Wang et al., 2020). With the recently released genome of *Thinopyrum elongatum*, in this study we analyzed the transcriptional reprogramming of six wheat-*Thinopyrum* substitution lines. Our results clearly showed opposite gene expression patterns between donor and recipient genomes, which are involved in gene silencing and genetic compensation, respectively. Interestingly, co-expression analysis of the orthologs supports genetic compensation from both the recipient and donor plants.

## Materials and Methods

### Sample Preparation and Sequencing

The plant materials used in this study comprised Tel (2*n* = 2*x* = 14, EE), CS wheat (2*n* = 6*x* = 42, AABBDD), and six disomic substitution (DS) lines, namely, DS3E(3A), DS3E(3B), DS3E(3D), DS7E(7A), DS7E(7B), and DS7E(7D). All samples were raised under a 16 h photoperiod and a 22°C temperature. The samples were harvested at three leave stages, then snap-frozen in liquid nitrogen, and held at −80°C. Three biological replicates were collected for each sample.

A total amount of 1 μg RNA per sample was used as the input material for the RNA sample preparations. Sequencing libraries were generated using the NEBNext^®^ Ultra™ RNA Library Prep Kit for Illumina (NEB, United States) following manufacturer’s recommendations, and index codes were added to attribute sequences to each sample. To select cDNA fragments of preferentially 150–200 bp in length, the library fragments were purified using the AMPure XP system (Beckman Coulter, Beverly, United States). PCR products were purified (AMPure XP system), and library quality was assessed on the Agilent Bioanalyzer 2100 system. The clustering of the index-coded samples was performed on a cBot Cluster Generation System using the TruSeq PE Cluster Kit version 3-cBot-HS (Illumina) according to the manufacturer’s instructions. After cluster generation, the library preparations were sequenced on an Illumina Hiseq platform, and 150 bp paired-end reads were generated.

### RNA-seq Analysis

Each of the reads for each of the three samples of Tel was mapped against the Tel genome, and CS samples were mapped against the wheat genome (IWGSC version 1.0) using TopHat (version 2.0.12) software (Kim et al., 2013; Appels et al., 2018; Wang et al., 2020). HTSeq version 0.6.1 was used to count the read numbers mapped to each gene (Anders et al., 2015). Then, Fragments Per Kilobase Per Million (FPKM) of each gene was calculated based on the length of the gene and reads count mapped to this gene. Transcripts recording a count of less than one per million mapped reads were ignored. The assignment of differentially expressed genes (DEGs) was based on two comparisons, i.e., the alien genes in DS lines compared with Tel and wheat genes except E chromosome in DS lines compared with CS. Differential expression analysis of two groups was performed using the DESeq R package (1.18.0). Genes with an adjusted *p*-value < 0.05 [false discovery rate (FDR)] found by DESeq were assigned as differentially expressed, and the Benjamini-Hochberg (BH) method was used for FDR control (Benjamini and Hochberg, 1995). The density of the transcribed genes (TGs) was calculated on the basis of a sliding 10 Mbp window. Similarly, the ratios of downregulated and upregulated to transcribed transcripts [R(Down/Trans), R(Up/Trans)] were calculated.

### Cytogenetic Analysis

The protocol for FISH was adopted as previously described (Yang et al., 2005). The seeds were germinated on a wet filter paper at room temperature (22°C), and the root tips were removed when the roots grew to 1.5–2.0 cm. The synthetic oligonucleotide probes, namely, oligo-PSC119.2 and oligo-PTA535 (Invitrogen Biotechnology, Inc.), were 5′ end-labeled green with 6-carboxyl fluorescein (6-FAM) or red with 6-carboxyl-tetramethylrhodamine (Tamra). The 6-μl probe system (20 ng/μl, 2 × SSC and 1 × TE buffer, pH 7.0) was denatured in boiling water for 5 min and then placed on ice. Then, hybridization experiments were carried out in a humid incubator at 37°C overnight. The slides were rinsed with 2 × SSC and treated with a VECTASHIELD mounting medium containing 1.5 μg/ml 4,6-diamidino-2-phenylindole (DAPI; Vector Laboratories). Images were captured using an Olympus BX-51 microscope equipped with a DP-70 CCD camera. Then, specific alien chromosomes were identified by comparing signal patterns.

### Identification of Orthologous Gene

The coding sequences (CDSs) of the two genomes were aligned reciprocally in a BLASTN search with the cutoff *E*-value of 1*e*^–10^. Gene pairs in the two compared genomes that have more than 80% sequence identity and greater than 60% of the total length of the orthologous region of the CDSs are regarded as orthologous genes.

### Functional Annotations and Enrichment Analysis

Protein sequences of CS and Tel were conducted using BLASTP search (*E*-value ≤ 10^–5^) against NCBI non-redundant (NR), Pfam, SwissProt, Kyoto Encyclopedia of Genes and Genomes (KEGG), and Gene Ontology (GO) databases (Chen et al., 2021). In addition, the omicShare website^1^ was used to perform the GO and KEGG enrichment. Hypergeometric test was used for statistical significance tests (*p* < 0.05).

## Results

**FIGURE 1:**
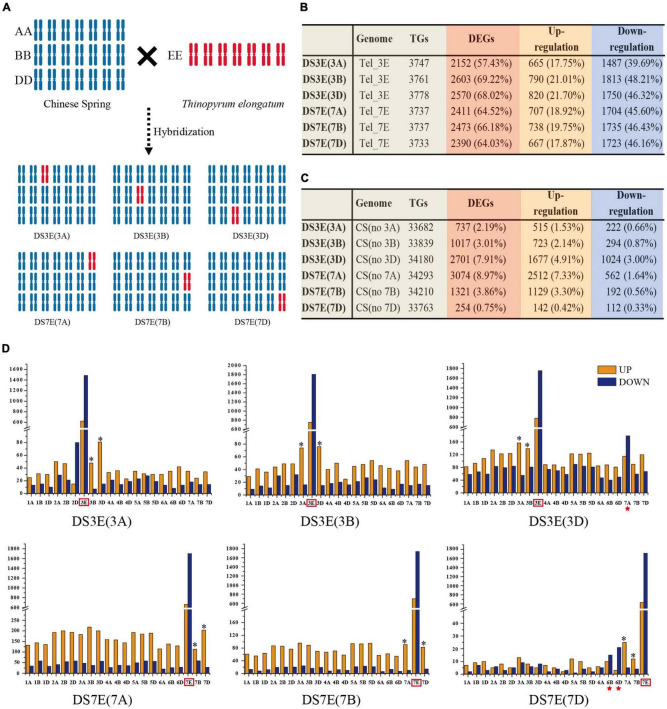
Detailed information on the differential expression of the transcriptome of the substitution line. **(A)** Schematic diagram of disomic substitution lines produced by distant hybridization between CS and Tel. **(B)** The differential transcription of Tel genes. **(C)** The differential transcription of CS genes. **(D)** Number of differentially expressed genes (DEGs) in alien Tel chromosome and CS genome. The alien Tel chromosome was surrounded by the red box. Homologous chromosomes from CS that belong to the same linkage group as the alien Tel chromosome were marked with an asterisk (*). The upregulated and downregulated genes were represented by the orange and blue rectangles. The chromosomes with missing fragments were marked in a red star.

The six *Triticum aestivum*-*T. elongatum* DS lines were previously generated by hybridization of hexaploid bread wheat cultivar “Chinese Spring” (CS, 2*n* = 6*x* = 42, AABBDD) with its diploid wild relative *T. elongatum* (Tel, 2*n* = 2*x* = 14, EE) (Figure 1A). FISH assays confirmed the substitutions in each of these lines (Supplementary Figure 1). To identify transcriptomic changes associated with the introgression of the 3E and 7E chromosomes into the wheat genome, we conducted RNA-seq analysis to compare each of the six substitution lines with both CS and Tel parents. An average of 40,698,776 clean reads for each library was generated (Supplementary Table 1).

### “Transcriptomic-Shock” for Exotic and Host Genomes

Reads of Tel samples were mapped against the Tel genome, and CS samples were mapped against the wheat genome (IWGSC version 1.0). Reads of the DS lines were mapped to the chimeric reference genomes according to the substituted sub-genome (Figure 1A). For example, the reference genome for DS3E(3D) is comprised of all the wheat sub-genomes but with chr. 3D replaced by chr. 3E. To determine whether substitution with Tel chromatin resulted in major transcriptomic changes compared with that of CS or Tel, we then calculated the expression levels (FPKM > 1) of each gene in the sequencing reads (i.e., TGs) and then identified the differentially expressed genes (DEGs) and non-DEGs (Tel genes in DS lines compared with Tel and wheat genes except E chromosome in DS lines compared with CS). The results showed that the frequency of DEGs for alien 3E and 7E chromosomes (57.43–69.22%) is much higher than that for wheat recipient genome (2.19–8.97%), which supported substantially stronger “transcriptomic-shock” for alien genes compared with genes from the CS recipient genome (Figure 1B). Notably, only 254 (0.75%) wheat DEGs were observed in comparisons of DS7E(7D) vs. CS (Figure 1C), indicating that chr. 7D substitution induced a very low degree of “transcriptomic-shock,” which is favorable for breeding program when engineering this chromosome.

### Distribution of Differentially Expressed Genes on E Genome

For all the six substitution lines, we found that the E chromosomes showed substantially downregulated DEGs (1,487–1,813 or 39.69–48.21%) than upregulated DEGs (665–820 or 17.75–21.70%) (Figure 1B and Supplementary Figure 2), implying that the alien genes were suppressed to a greater extent than native genes following substitution. This suppressive effect could be detected along the full length of each substituted E chromosome. In addition, both the number and ratio of downregulated DEGs were higher in the distal regions of the short and long arms, while the downregulated or upregulated DEGs tended to localize near the centromeres (Supplementary Figures 3A,B). Moreover, most of the upregulated or downregulated DEGs were the same genes regardless of which sub-genome was substituted and were true for both chr. 3E and chr. 7E (Supplementary Figure 3C and Supplementary Table 2), suggesting that a similar underlying mechanism was responsible for this effect across genes and chromosomes.

### Transcriptional Compensation in Wheat

In comparison with alien genes from the E genome, the DEGs from wheat showed an opposite expression pattern that upregulated genes were apparently more abundant than downregulated in all DS lines (Figure 1D), which suggested genetic compensation for the substitution of either chr. 3 or chr. 7. This compensation could be detected on almost all the endogenous wheat chromosomes, even the corresponding homeologous chromosomes of 3A, 3B, 3D or 7A, 7B, 7D showed a relatively higher amount of upregulated genes than downregulated genes (Figure 1D). It is noted that, when chr. 7E substituted its D sub-genome homolog, the DEGs were relatively fewer than that under A and B sub-genome substitutions (Figure 1D), suggesting weaker transcriptomic influence for loss of the chr. 7D. Moreover, even most wheat endogenous sub-genomes exhibited more upregulated genes suggesting that genetic compensation, chr. 7A in DS3E(3D), and chr. 6B and chr. 6D in DS7E(7D) showed more downregulated genes (Figure 1D), which might be due to the potential fragment deletion on these chromosomes. No significant expression was detected for the genes located at the most distal region of Chr. 7AL (∼36 Mb) in the substitution line of DS3E(3D), indicating a chromosome fragment loss of the recipient genome (Supplementary Figure 4 and Supplementary Table 3). Similarly, potential loss of genome fragments from the distal ends of Chr. 6B and Chr. 6D was also observed in the DS7E(7D) substitution line (Supplementary Figure 5 and Supplementary Table 3).

### Co-expression of Orthologs Between E and Wheat Sub-Genomes

**FIGURE 2:**
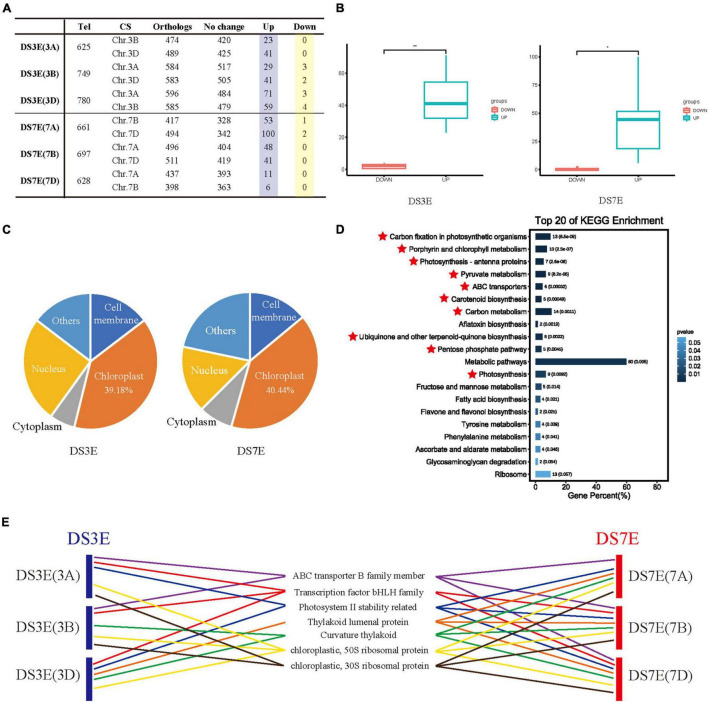
Transcriptional changes and functional analysis of CS homeologous genes in DS3E and DS7E. **(A)** The differential transcription of CS orthologs of upregulated genes in DS3E and DS7E. **(B)** Comparison of upregulated and downregulation of CS homologous genes. Statistical analysis was performed using the *T*-test method. *p < 0.05. **p < 0.01. **(C)** Subcellular localization of CS homologous genes. **(D)** Kyoto Encyclopedia of Genes and Genomes (KEGG) enrichment of wheat homologous genes. The photosynthesis-related genes with *p* < 0.01 were marked by the red star. **(E)** Distribution of photosynthesis-related genes in different substitution lines. Different items were represented by different color lines. The same item was represented by a line of the same color between different substitution lines.

Incomplete compensation is generally presumed to occur between the different homeologous sub-genomes of wheat (Leach et al., 2014). However, in our study, we found that the compensatory transcriptional effects following chromosome substitution were detectable across homeologous chromosomes from the intact sub-genomes as well as in almost all the other wheat chromosomes (Figure 1D). To investigate potential correlations between the transcription of orthologous donor and recipient genes, we compared the DEGs from the E genome with their orthologs in the wheat sub-genome (e.g., the orthologs on chr. 3B and chr. 3D were studied in the DS3E(3A) substitution line). The results showed that a dozen of wheat orthologs that corresponded to upregulated alien genes were also transcriptionally upregulated, while few CS orthologs showed downregulation (Figures 2A,B, Supplementary Figure 6, and Supplementary Tables 4, 5). For example, among the 665 upregulated exotic genes from DS3E(3A), 23 and 41 orthologs were upregulated on chr. 3B and chr. 3D, respectively. In contrast, the number of downregulated orthologs from the chr. 3B and D sub-genomes was 0 (Figure 2A). This co-expression pattern for upregulated genes implied that the observed compensation for chromatin which was lost from wheat was potentially attributable to both donor and recipient genomes.

To further investigate the possible underlying mechanisms, we submitted all the co-upregulated orthologous genes (429) of wheat to the Cell-PLoc version 2.0 website^2^ to predict their subcellular localization (Supplementary Table 6). Interestingly, most of these genes were expressed in the chloroplast, from both 3E (39.18%) and 7E (40.44%), which implied their involvement in photosynthesis (Figure 2C and Supplementary Table 6). We also examined the annotation of these co-upregulated orthologs using the KEGG, Swissprot, Pfam, and NCBI nr databases (Supplementary Tables 7, 8). Subsequent KEGG enrichment analysis showed that the co-expressed genes were mainly enriched in pathways for photosynthesis-related functions, such as “Carbon fixation,” “Porphyrin and chlorophyll metabolism,” and “Pyruvate metabolism” among others (Figure 2D and Supplementary Figure 5). Detailed analysis of their functional annotations revealed a large number of genes among the upregulated DEGs with the same or similar functions, for example, encoding thylakoid lumenal proteins, NAD(P)H, 30S, and 50S chloroplast ribosomal proteins, which directly participate in photosynthetic processes (Figure 2E and Supplementary Tables 7–9; Liu and Last, 2017; Pulido et al., 2018; Ma et al., 2021). Moreover, many bHLH transcription factors and ABC transporter genes were also enriched, which are not expressed in the chloroplast but are widely reported to contribute to photosynthesis (Figure 2E and Supplementary Tables 7–9; He et al., 2020; Yu et al., 2021), which further support that the co-upregulated gene cluster is involved in the photosynthesis process. Thus, the co-expression of these genes in compensation for lost chromosomes may imply defects in photosynthesis of distant hybridization products, especially for substitution lines.

## Discussion

Distant hybridization, the practice crossing two different species or closely related genera, has been proven successful as a strategy for introducing agronomically beneficial genes from wild species into cultivated crops to increase their yield, quality, and resistance to biotic and abiotic stresses (Moore et al., 2006; Endo, 2007). In wheat, efforts to create useful introgression lines remain ineffective, either due to linkage drag, incomplete genetic compensation, or our relatively poor understanding of “transcriptomic-shock” related to distant hybridization (Buggs et al., 2011). In this study, RNA-seq analysis was used to analyze transcriptomic changes in six wheat-*Thinopyrum* substitution lines compared with that in their wild-type genetic backgrounds.

Our results clearly showed substantially stronger transcriptomic-shock for alien genes compared with genes from the CS recipient genome, with as much as 57.43–69.22% of E genome-derived genes exhibiting significantly different expressions from that in *T. elongatum* (Figure 1B and Supplementary Figure 2). This finding was consistent with previous results observed in a barley-wheat addition line (Rey et al., 2018). Similar findings also showed that the number of downregulated alien DEGs was far greater than the number of upregulated DEGs. This suppressive effect on alien gene expression could at least partially explain the frequently observed loss of function among disease resistance genes following transfer by distant hybridization into wheat from other *Triticeae* species. The mechanisms underlying large-scale transcriptomic changes are presumably complex, involving both genetic and epigenetic components. It is reasonable to speculate that the transcriptional machinery required for control of alien genes is absent or incompatible with that in the wheat genetic background, e.g., transcription factors do not fully match with the targeted *cis*-regulatory elements in alien chromatin (Li et al., 2019). A recent analysis of three-dimensional chromatin architecture in allohexaploid wheat variety Aikang 58, which carries the 1RS/1BL translocation chromosome, showed a low frequency of inter-chromosomal interactions involving the translocated 1RS chromosomal arm (Jia et al., 2021).

In contrast with alien genes, many fewer genes native to wheat were differentially expressed (4.45% average), but those DEGs significantly differed between substitution lines. Only 254 (0.75%) of the native wheat genes were differentially expressed in the DS7E(7D) line, which suggested that chromosome engineering of 7D could serve as a potentially effective strategy for introducing beneficial genes from wild relatives with low impact on the host plant (Figure 1C). Moreover, we observed strong apparent transcriptional compensation, based on the significantly greater number of upregulated DEGs in wheat compared with the number of downregulated DEGs (Figure 1D). We found that the compensatory transcriptional effects almost all the wheat chromosomes not just incomplete compensation supported by a study using nullisomic-tetrasomic wheat lines (Zhang et al., 2019). Notably, the corresponding homologous chromosomes showed a higher proportion of upregulated DEGs than other chromosomes (Figure 1D).

In addition, substitution lines in which the 7D sub-genome was replaced, DS7E(7D), showed a lower ratio of upregulated genes compared with those with replacement of the A and B sub-genomes. This finding indicated relatively weaker compensation for the 7D sub-genome, although the underlying mechanisms require further investigation to unravel. Interestingly, we found small genome fragment deletions in these two D sub-genome substitution lines on the distal ends of chr. 7AL, chr. 6BL, and chr. 6DL (Supplementary Figures 4, 5 and Supplementary Table 3). This fragment deletion on chr. 7AL was also observed in another barley-wheat addition line, and these deletions were possibly caused by chromosomal rearrangements that occurred during the hybridization process, given that wheat-barley hybridization has been reported to trigger structural changes and chromosomal instability (Rey et al., 2018).

Another important finding of this study is that many orthologs of the upregulated alien genes are also upregulated in wheat, indicating that the genetic impact caused by chromosome substitution can be compensated by both the donor and recipient genomes (Figures 2A,B and Supplementary Figure 6). We found that the striking functional redundancy of these co-expressed genes was related to photosynthesis, a fundamental biological process in plants. The majority of these genes were predicted to be localized in the chloroplast (Figure 2C and Supplementary Table 6), and even bHLH transcription factors and ABC transporter among these co-expressed genes are known to contribute to photosynthesis (Dong et al., 2014; Borba et al., 2018; Voith von Voithenberg et al., 2019; Li et al., 2020), despite predicted subcellular localization to other compartments. For instance, overexpression of *MdbHLH3* can increase photosynthetic capacity and carbohydrate levels of apple leaves, thereby enhancing the accumulation of carbohydrates in fruit (Yu et al., 2021). *OsABCI7* can interact with *OsHCF222* to stabilize the thylakoid membrane in rice (He et al., 2020). Photosynthesis is a major determinant of crop yield and represents a central target for breeding improvement of quantitative agronomic traits. Thus, the co-expression of these genes in compensation for lost chromosomes could imply that the often poor performance of yield traits by distant hybridization lines, especially substitution lines, may be attributable to defects in photosynthesis, as well as the linkage drag revealed in previous studies (Mondal et al., 2016).

## Conclusion

This is the first finding on transcriptional compensation in wheat-*Thinopyrum* substitution lines. Our research indicated that both the alien genes derived from Tel and CS endogenous genes compensate the replaced chromosome, and this finding explains the feasibility of distant hybridization in some extent, which provided theoretical reference for the application of related species in wheat breeding.

## Data Availability Statement

The original contributions presented in the study are publicly available. This data can be found here: National Center for Biotechnology Information (NCBI) BioProject database under accession number PRJNA789995.

## Author Contributions

HWW and LK designed and supervised the manuscript. ZL, SX, HJW, ML, WG, BH, XC, XL, NC, TZ, YB, and ZY performed the research. YH, LC, SS, and JJ analyzed the data. ZL and YH wrote the manuscript. All authors read and approved the final manuscript.

## Conflict of Interest

LC was employed by the company Smartgenomics Technology Institute. The remaining authors declare that the research was conducted in the absence of any commercial or financial relationships that could be construed as a potential conflict of interest.

## Publisher’s Note

All claims expressed in this article are solely those of the authors and do not necessarily represent those of their affiliated organizations, or those of the publisher, the editors and the reviewers. Any product that may be evaluated in this article, or claim that may be made by its manufacturer, is not guaranteed or endorsed by the publisher.

## Footnotes

1
https://www.omicshare.com/tools/Home/Soft/getsoft

2
http://www.csbio.sjtu.edu.cn/bioinf/Cell-PLoc-2/

## Supplementary Material

Supplementary Figure 1 Karyotypes of the six disomic substitution (DS) lines in the FISH assay, and the red arrow represents the alien 3E and 7E chromosomes.

Supplementary Figure 2 Comparison of the number of upregulated genes and downregulated genes in DS3E and DS7E. Upregulated and downregulated genes were represented by different colors. Statistical analysis was performed using the *T*-test method. ^****^*p* < 1*e*-5.

Supplementary Figure 3 Distribution of the differentially expressed genes (DEGs) and non-DEGs along the 3E and 7E chromosomes. **(A)** Distribution in the number of the DEGs and non-DEGs along the 3E and 7E chromosomes. **(B)** Distribution in the proportion of the DEGs and non-DEGs along the 3E and 7E chromosomes. **(C)** Overlapped DEGs of DS lines belonging to the same linkage group.

Supplementary Figure 4 The chromosomal spread of 7A genes showing altered transcription in DS3E(3D). The blue shadow in the 7AL terminal represents the deletion region of the wheat genome. The ratio of not-DT to transcribed genes (TGs) [R(not-DT/Trans)], the ratio of upregulated to TGs [R(Up/Trans), the ratio of downregulated to TGs [R(Down/Trans)], and the mean log FC in CS along the 7A chromosome were represented by different colors.

Supplementary Figure 5 The chromosomal spread of 7A genes showing altered transcription in DS7E(7D). **(A)** Deletion of the chromosomal fragment that occurs on 6BL. **(B)** Deletion of the chromosomal fragment that occurs on 6DL. The blue shadow represents the deletion region. The ratio of not-DT to TGs [R(not-DT/Trans)], the ratio of upregulated to TGs [R(Up/Trans), the ratio of downregulated to TGs [R(Down/Trans)], and the mean log FC in CS along the 6B and 6D chromosomes were represented by different colors.

Supplementary Figure 6 Comparison of upregulation and downregulation of homologous genes of downregulated genes in 3E and 7E. Statistical analysis was performed using the *T*-test method. *NS*, no significant.
