# Supplementary figures and images for: Wheat-Thinopyrum Substitution Lines Imprint Compensation Both From Recipients and Donors
Source: Front Plant Sci. 2022 Apr 15;13:837410. doi: 10.3389/fpls.2022.837410 (PMC9051513; doi:10.3389/fpls.2022.837410)

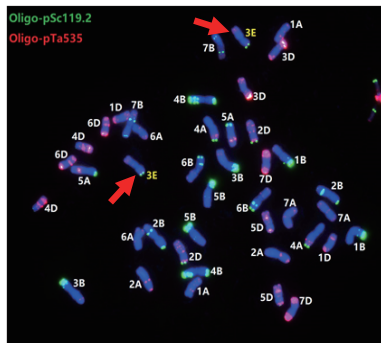

DS3E(3A)

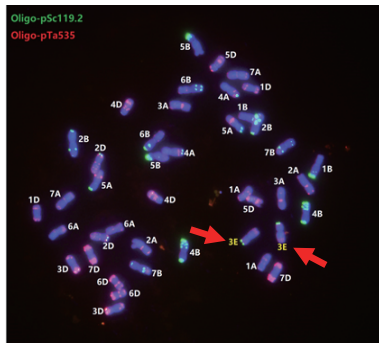

DS3E(3B)

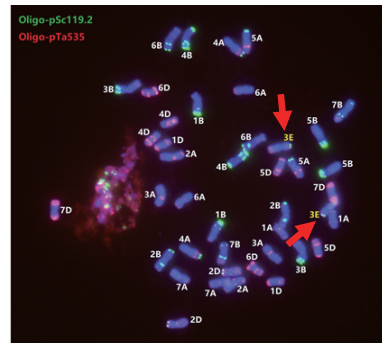

DS3E(3D)

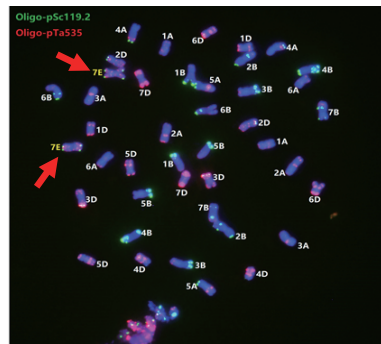

DS7E(7A)

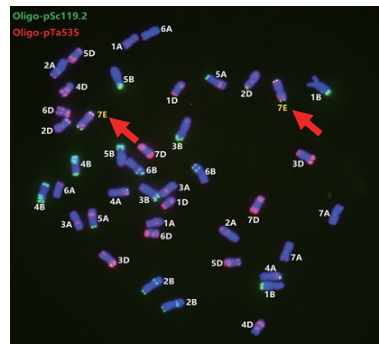

DS7E(7B)

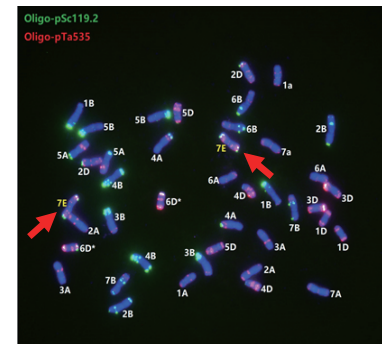

DS7E(7D)

Supplement: Supplementary Figure 1 — Karyotypes of the six disomic substitution (DS) lines in the FISH assay, and the red arrow represents the alien 3E and 7E chromosomes. [file Image_1.PDF]

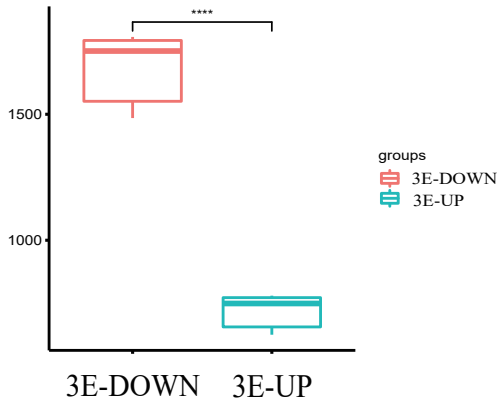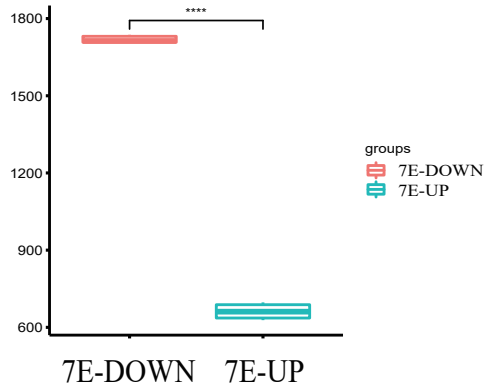

Supplement: Supplementary Figure 2 — Comparison of the number of upregulated genes and downregulated genes in DS3E and DS7E. Upregulated and downregulated genes were represented by different colors. Statistical analysis was performed using the T-test method. ****p < 1e-5. [file Image_2.PDF]

A

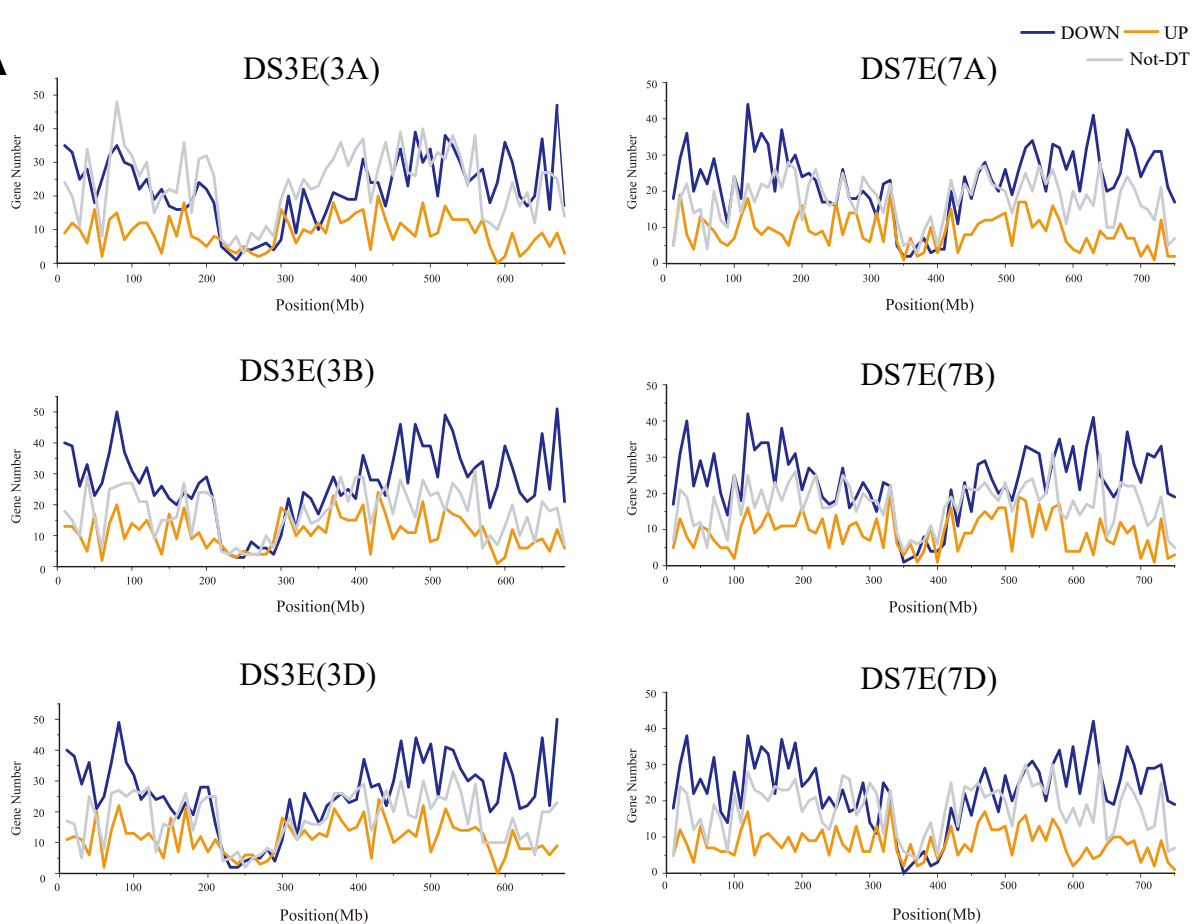

B

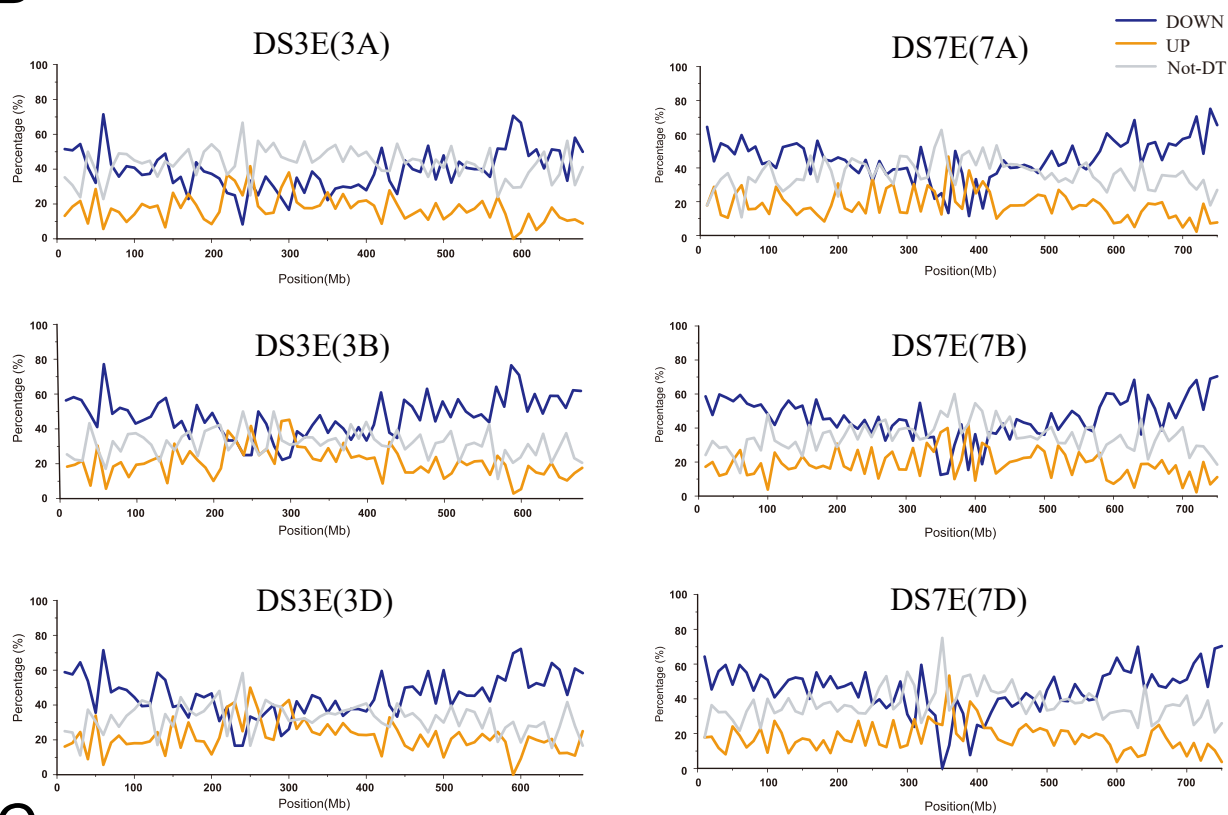

C

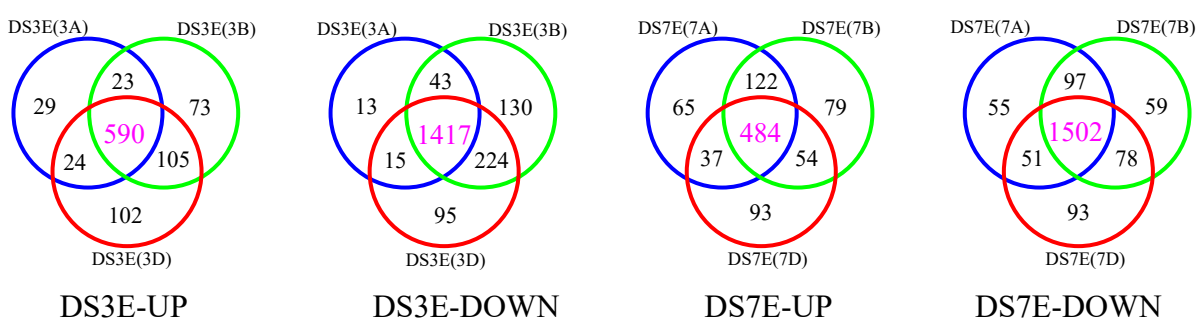

Supplement: Supplementary Figure 3 — Distribution of the differentially expressed genes (DEGs) and non-DEGs along the 3E and 7E chromosomes. (A) Distribution in the number of the DEGs and non-DEGs along the 3E and 7E chromosomes. (B) Distribution in the proportion of the DEGs and non-DEGs along the 3E and 7E chromosomes. (C) Overlapped DEGs of DS lines belonging to the same linkage group. [file Image_3.PDF]

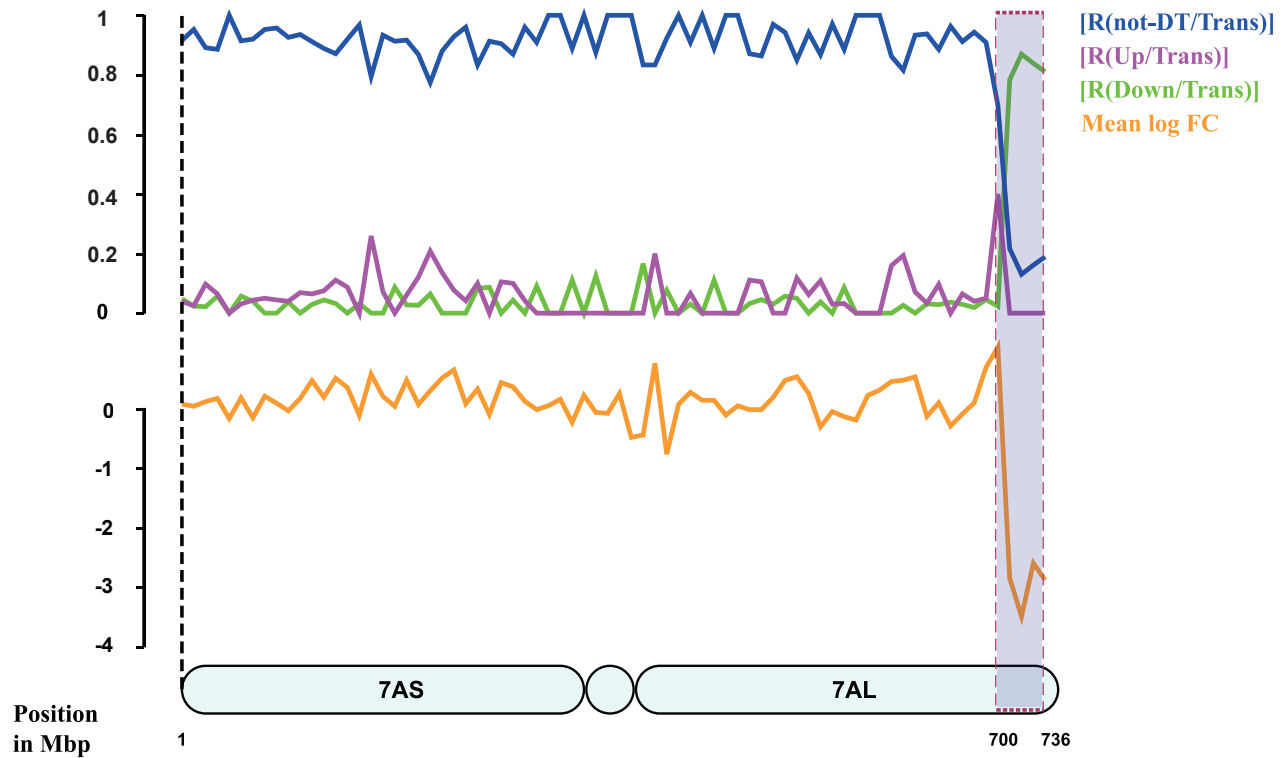

Supplement: Supplementary Figure 4 — The chromosomal spread of 7A genes showing altered transcription in DS3E(3D). The blue shadow in the 7AL terminal represents the deletion region of the wheat genome. The ratio of not-DT to transcribed genes (TGs) [R(not-DT/Trans)], the ratio of upregulated to TGs [R(Up/Trans), the ratio of downregulated to TGs [R(Down/Trans)], and the mean log FC in CS along the 7A chromosome were represented by different colors. [file Image_4.PDF]

A

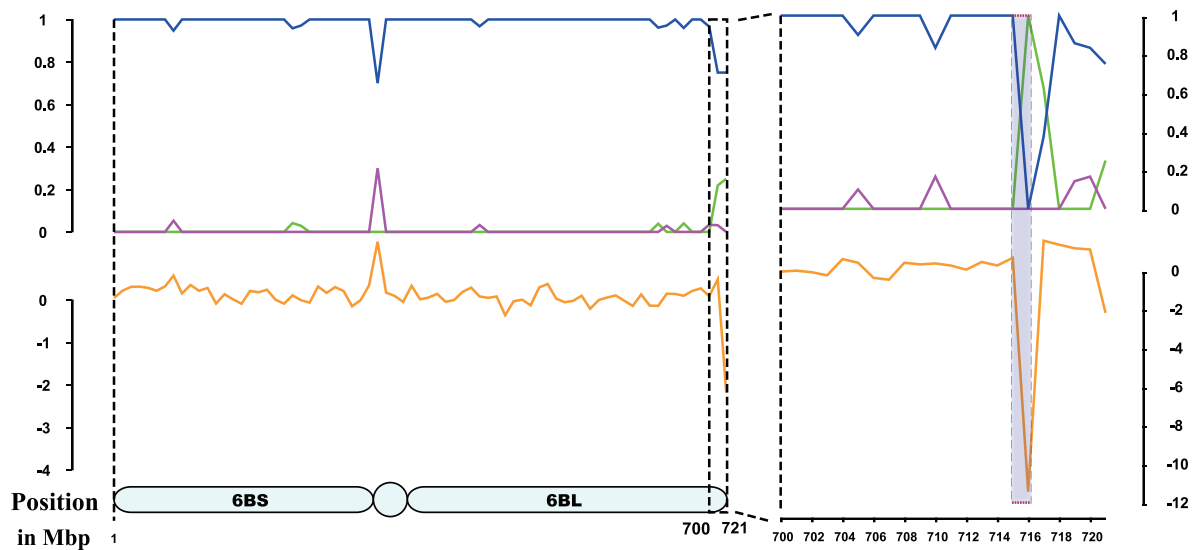

B

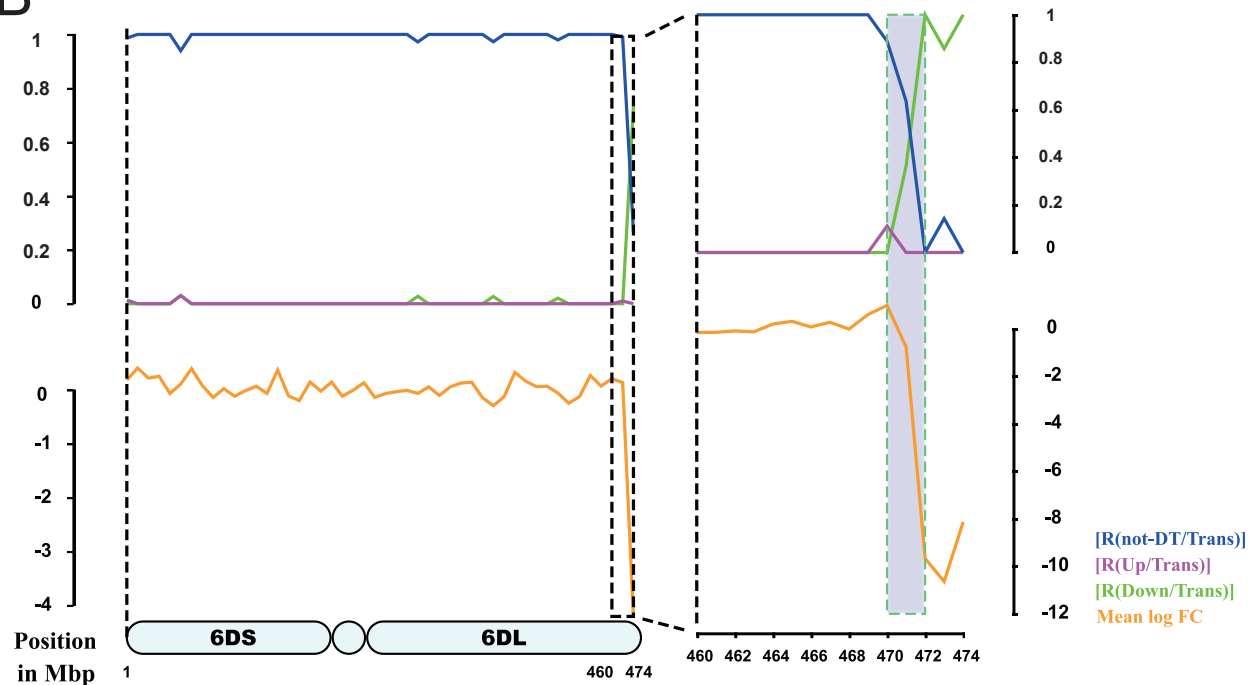

Supplement: Supplementary Figure 5 — The chromosomal spread of 7A genes showing altered transcription in DS7E(7D). (A) Deletion of the chromosomal fragment that occurs on 6BL. (B) Deletion of the chromosomal fragment that occurs on 6DL. The blue shadow represents the deletion region. The ratio of not-DT to TGs [R(not-DT/Trans)], the ratio of upregulated to TGs [R(Up/Trans), the ratio of downregulated to TGs [R(Down/Trans)], and the mean log FC in CS along the 6B and 6D chromosomes were represented by different colors. [file Image_5.PDF]

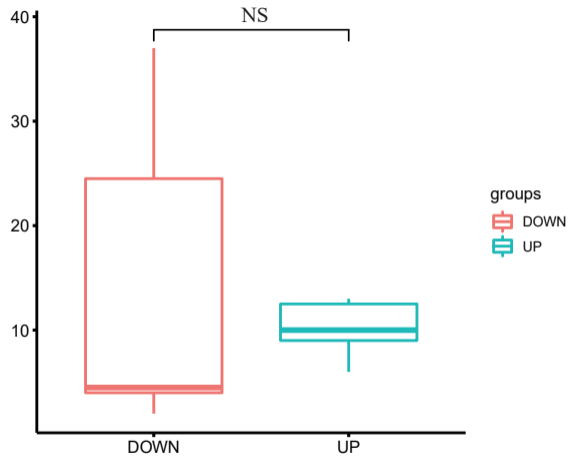

DS3E

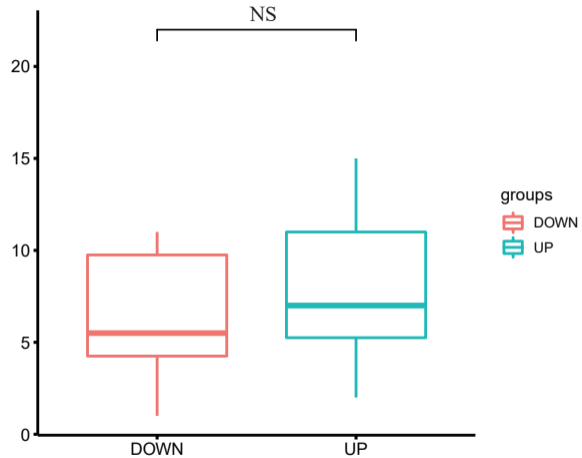

DS7E

Supplement: Supplementary Figure 6 — Comparison of upregulation and downregulation of homologous genes of downregulated genes in 3E and 7E. Statistical analysis was performed using the T-test method. NS, no significant. [file Image_6.PDF]
